# Maternal Mortality in Taiwan: A Nationwide Data Linkage Study

**DOI:** 10.1371/journal.pone.0132547

**Published:** 2015-08-03

**Authors:** Tung-Pi Wu, Fu-Wen Liang, Ya-Li Huang, Lea-Hua Chen, Tsung-Hsueh Lu

**Affiliations:** 1 Department of Obstetrics and Gynecology, Sin-Lau Christian Hospital, Tainan, Taiwan; 2 NCKU Research Center for Health Data and Department of Public Health, National Cheng Kung University, Tainan, Taiwan; 3 Department of Public Health, School of Medicine, College of Medicine, Taipei Medical University, Taipei, Taiwan; 4 Department of Statistics, Ministry of Health and Welfare, Taipei, Taiwan; Centers for Disease Control, TAIWAN

## Abstract

**Background:**

To examine the changes in the maternal mortality ratio (MMR) and causes of maternal death in Taiwan based on nationwide linked data sets.

**Methods:**

We linked four population-based data sets (birth registration, birth notification, National Health Insurance inpatient claims, and cause of death mortality data) to identify maternal deaths for 2004–2011. Subsequently, we calculated the MMR (deaths per 100,000 live births) and the proportion of direct and indirect causes of maternal death by maternal age and year.

**Findings:**

Based on the linked data sets, we identified 236 maternal death cases, of which only 102 were reported in officially published mortality data, with an underreporting rate of 57% [(236−102) × 100 / 236]. The age-adjusted MMR was 18.4 in 2004–2005 and decreased to 12.5 in 2008–2009; however, the MMR leveled off at 12.6 in 2010–2011. The MMR increased from 5.2 in 2008–2009 to 7.1 in 2010–2011 for patients aged 15–29 years. Women aged 15–29 years had relatively lower proportion in dying from direct causes (amniotic fluid embolism and obstetric hemorrhage) compared with their counterpart older women.

**Conclusions:**

Approximately two-thirds of maternal deaths were not reported in officially published mortality data. Routine surveillance of maternal mortality by using enhanced methods is necessary to monitor the health status of reproductive-age women. Furthermore, a comprehensive maternal death review is necessary to explore the preventability of these maternal deaths.

## Introduction

The maternal mortality ratio (MMR) is a crucial indicator of the health status of reproductive-age women [1]. The fifth goal of the Millennium Development Goals, established by the United Nations in 2000, was to reduce the MMR by 75% between 1990 and 2015 [2]. Accurate MMR estimation is essential in planning relevant programs to reduce the MMR. However, despite the availability of complete vital registration systems in high-income countries, the MMRs reported in the officially published mortality data remain underreported [3–13]. For example, the number of maternal deaths reported in the Confidential Enquiry into Maternal Deaths program in the United Kingdom was 60% higher than that in the routine vital registration system [13].

Why were the maternal deaths of official published mortality data in most countries underreported? Because official published mortality data were based on information recorded on the death certificate only. Medical certifiers might intentionally (to avoid legal suits) or unintentionally (to report only the mechanism of death) omit the pregnancy-related information on the death certificate. For example, women died from amniotic fluid embolism or postpartum hemorrhage might often been recorded as respiratory failure or disseminated intravascular coagulopathy on the death certificate. The underlying cause of death would be attributed to disease of respiratory system (Chapter X in International Disease of Classification Tenth Revision, ICD-10) or disease of blood or blood forming organ (Chapter III in ICD-10) instead of pregnancy, childbirth and the puerperium (Chapter XV in ICD-10).

The most commonly used method to assess the extent of MMR underreporting is to link the death certificates of reproductive-age women to their birth or fetal death certificates [5–8,10–12]. In some states in the United States, the surveillance of maternal mortality has been further enhanced by matching birth and fetal death certificates to the hospital discharge data, medical examiners’ records, and any other available sources [7,8,10].

Relatively few studies from middle-income countries have linked nationwide data sets to estimate the MMR [14,15]. A study from Jamaica reported that only 12 of 50 maternal deaths (identified by investigators) had been coded as maternal deaths in the officially registered mortality data [15]. However, their analysis was confined to 1 year. A recent study from Taiwan using sampled data (2 million of the population of 23 million) linked to the National Health Insurance (NHI) inpatient claims data and cause of death (COD) mortality data reported that approximately two-thirds of the identified maternal deaths were not reported in the officially published mortality data [16]. However, because few maternal deaths were identified in that study, they could not examine the changes in the MMR or CODs. Therefore, the purpose of our study was to link data among the four nationwide population-based data sets in Taiwan—birth registration (BR), birth notification (BN), NHI inpatient claims, and COD—to examine the changes in the MMR and causes of maternal death from 2004 to 2011.

## Materials and Methods

### Background information on Taiwan

In 2012, Taiwan had a population of 23 million and a gross national income per capita of US$21,967, which was lower than that of South Korea (US$30,178) and Japan (US$36,752), two countries with a similar Confucian background [17,18]. The total fertility rate in 2014 was 1.11 in Taiwan, 1.25 in South Korea, and 1.42 in Japan [19]. Despite the decrease in the number of childbirths in the past two decades, (from 325,994 live births in 1993 to 194,939 live births in 2013), the proportion of older reproductive-age women (35−49 years) among all births increased from 5% in 1993 to 22% in 2013 [20]. The number of maternal deaths (ratio, deaths per 100,000 live births) according to official published cause of death statistics for years 2008 through 2012 was 13 (6.6), 16 (8.3), 7(4.2), 10(5.0), and 20(8.5), respectively [21].

### Data source

Table 1 lists the basic information of the four nationwide population-based data sets. In Taiwan, every resident is assigned an unique identification number that is recorded and maintained in the aforementioned administrative data sets, which can be used for record linkage. To facilitate the linkage of various government administrative data sets, the Center for Health and Welfare Data Analysis and Application was established by the Department of Statistics, Ministry of Health and Welfare, Taiwan [22]. To protect patient identity and privacy sets, the linkage analyses were conducted in an isolated, restricted-access room. Only aggregated statistical tables were released for research after careful inspection by the center staff to ensure that the results did not reveal personal information.

**Table 1:** Basic information of four almost complete coverage national data sets.

	Birth registration data	Birth notification data	Inpatient NHI claims data	Mortality data
**Government in charge**	the Department of Household Registration Affairs, Ministry of Internal Affairs	the Health Promotion Administration, Ministry of Health and Welfare	the National Health Insurance Administration, Ministry of Health and Welfare	the Department of Statistics, Ministry of Health and Welfare
**Reported by**	Parents of newborn to get birth certificate	All live and still births delivered in hospitals or clinics are reported by medical professionals.	Hosptial managers	Physicians, coroners, or medical examiners who issued death certificate
**Requirement**	Within 60 days after birth	Within 7 days after delivery	On average one month	Within 15 days after death
**Important variables**	Education atainment of parents, birthweight and gestation weeks	Birthweight and gestation weeks, type of delivery, fetal death, Apgar score of newborn	Five discharge diagnoses and procedures, medical care fee, characteristics of hospitals	Cause of death, manner of death, place of death
**Electronized data** *	Since 1979	Since 2001	Since 1996	Since 1971
**Quality of data**	Many foreign born mothers did not have ID, accuracy of birthweight and gestation weeks information fair	Many foreign born mothers did not have ID, accuracy of birthweight and gestation weeks information most reliable	The only data having pregnancy- related diagnosis, accuracy of birthweight and gestation weeks information fair	Certifiers might omit the pregnancy information intentionally or unintentionally

*Years available for linkage were 2004–2011.

### Identification of maternal death

A maternal death was defined as reproductive-age women (15–49 years) registered in COD mortality for years 2004 through 2011 and also registered in BR or BN for the same period. Because of some errors in registration of basic information, we further focused on deaths in which the birth and maternal death dates were within 42 days, according to the definition of maternal death established by the World Health Organization.

### Determining the cause of maternal death

To obtain more information to determine the cause of maternal death, we further linked to the NHI inpatient claims data set to get discharge diagnoses. We considered both discharge diagnoses (ICD-9-CM codes) registered in NHI inpatient claims and CODs (ICD-10 codes) recorded in the mortality to determine the causes of maternal death. Some cases had identical diagnoses in the two data sets, whereas the diagnoses for some cases differed between the two data sets because some medical certifiers might intentionally or unintentionally exclude pregnancy-related information while issuing a death certificate. For example, “amniotic fluid embolism” was recorded in the NHI inpatient claims data set, whereas “respiratory failure” was recorded as the underlying COD in mortality data set. In these discrepant cases, we determined the cause of maternal death according to the pregnancy-related discharge diagnoses recorded in the NHI claims data. If two or more pregnancy-related diagnoses were recorded in the NHI claims data, we selected the first-reported diagnosis.

Causes of maternal death were classified into two types: direct and indirect causes. Deaths resulting from obstetric complications, such as hypertension (ICD-9-CM code 642, ICD-10 codes O10-O16), amniotic fluid embolism (ICD-9-CM code 673, ICD-10 code O88), or postpartum hemorrhage (ICD-9-CM code 666, ICD-10 code O72), were defined as direct causes. Deaths resulting from a preexisting disease or a disease that developed during pregnancy and was not due to any of the direct obstetric cause but was aggravated by the physiological effects of pregnancy, such as infectious diseases (ICD-9-CM codes 001–139, ICD-10 codes A00–B99), neoplasm (ICD-9-CM codes 140–239, ICD-10 codes C00–D48), or circulatory system-related diseases (ICD-9-CM codes 390–459, ICD-10 codes I00–I99), were defined as indirect causes.

### Statistical analysis

First, we calculated the underreporting rate between the officially reported number of maternal deaths and those determined from the linked data. The underreporting rate (%) was calculated according to the following formula: (number of deaths estimated using the linked data–the number of officially reported deaths) × 100 / the number of deaths estimated using the linked data. Second, we computed the MMR (deaths per 100,000 live births) according to the maternal age and year. Because the proportion of reproductive-age women has markedly increased in the past two decades, we calculated the age-adjusted MMR according to the age groups of 2004–2011 combined as a standard population. Finally, we compared the proportions of direct and indirect causes of maternal death by maternal age and year.

The study design was approved by the Institutional Review Board of National Cheng Kung University Hospital (B-ER-102-120-t). The data in the administrative data sets used in our study were scrambled, and the researchers were provided identification numbers of those insured to protect patient privacy. Informed consent was not obtained because patient information was anonymized and de-identified prior to analysis.

## Results

**Fig 1:**
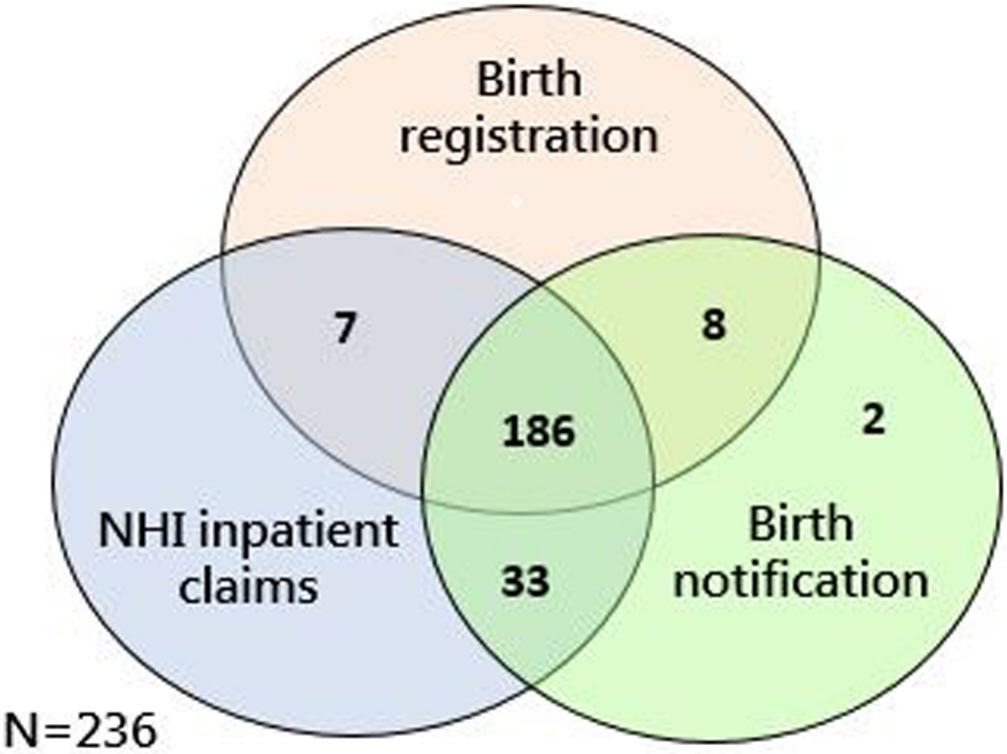
Number of identified maternal deaths from different data sets.

We initially identified 266 potential maternal death cases from the four linked data sets. Of these, the birth date was inaccurately recorded after the death date in seven cases, and incident deaths were reported in 23 cases (died of unintentional injuries, suicides, or homicides). After excluding these cases, we included 236 maternal death cases in the study. The cases from the different data sets are illustrated in Fig 1. There were 35 cases notified but not registered. On the contrary, there were 7 cases registered but not notified.

Of the 236 maternal deaths, only 102 were recorded in the officially published mortality data, with an average underreporting rate of 57% [(236−102) × 100 / 236]. The underreporting rates were similar for the different age groups: 58%, 58%, and 54% among women aged 15–29, 39–34, and 35−49 years, respectively. The underreporting rate decreased from 61% in 2004–2005 to 42% in 2008–2009 but increased to 65% in 2010–2011.

**Fig 2:**
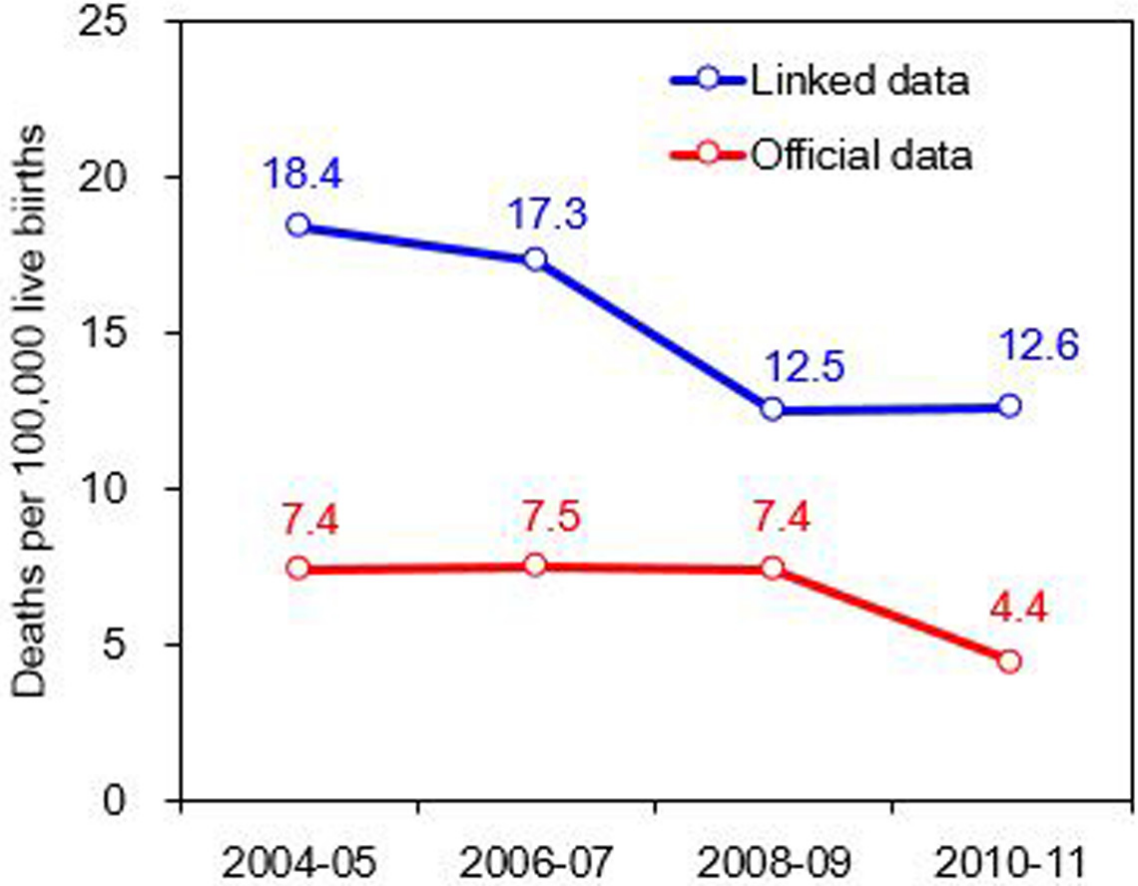
Age-adjusted maternal mortality ratios according to the linked data and officially published mortality data in Taiwan for 2004−2011.

**Fig 3:**
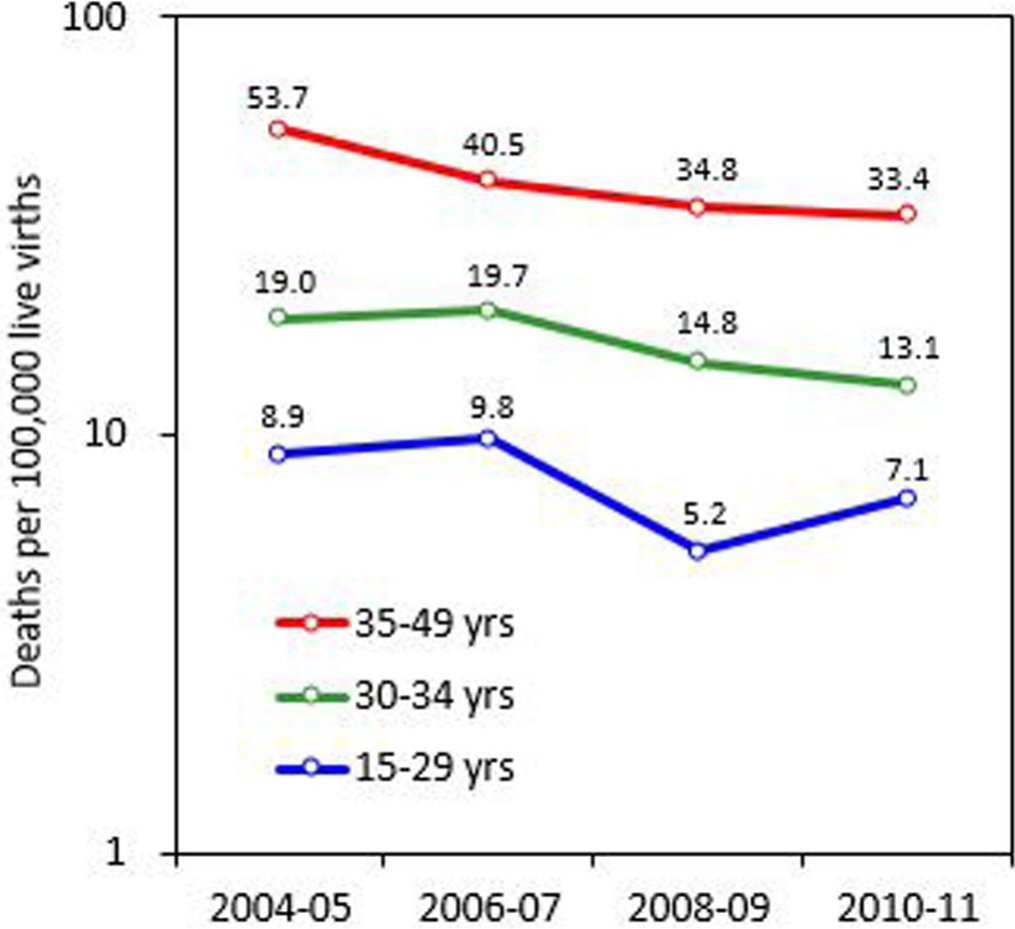
Maternal mortality ratios according to the reproductive age in Taiwan for 2004–2011.

The age-adjusted MMR was 18.4 in 2004–2005 and decreased to 12.5 in 2008–2009, but leveled off at 12.6 in 2010–2011. Conversely, according to the officially published mortality data, the age-adjusted MMR trends were stable from 2004–2005 to 2008–2009 and declined in 2010–2011 (Fig 2). Table 2 lists the age-specific MMR by year. An increase in the MMR from 5.2 in 2008–2009 to 7.1 in 2010–2011 was observed for women aged 15–29 years (Fig 3).

**Table 2:** Number of maternal deaths/births and maternal mortality ratios (deaths per 100,000 live births) according to the maternal age and year in Taiwan for 2004–2011 based on a nationwide data linkage study.

Maternal age	2004−05	2006−07	2008−09	2010−11	2004–11
	**Number of deaths/births**				
**15−29 years old**	23/257,099	22/225,289	10/190,634	10/141,835	65/814,857
**30−34 years old**	23/121,050	26/131,726	21/141,839	18/137,809	88/532,424
**35−49 years old**	24/44,664	20/49,387	19/54,606	20/59,954	83/208,611
**15−49 years old**	70/422,813	68/406,402	50/387,079	48/339,598	236/1,555,892
	**Maternal mortality ratio**				
**15−29 years old**	8.9	9.8	5.2	7.1	8.0
**30−34 years old**	19.0	19.7	14.8	13.1	16.5
**35−49 years old**	53.7	40.5	34.8	33.4	39.8
**15−49 years old** *	18.4	17.3	12.5	12.6	15.2

*Age−adjusted.

**Fig 4:**
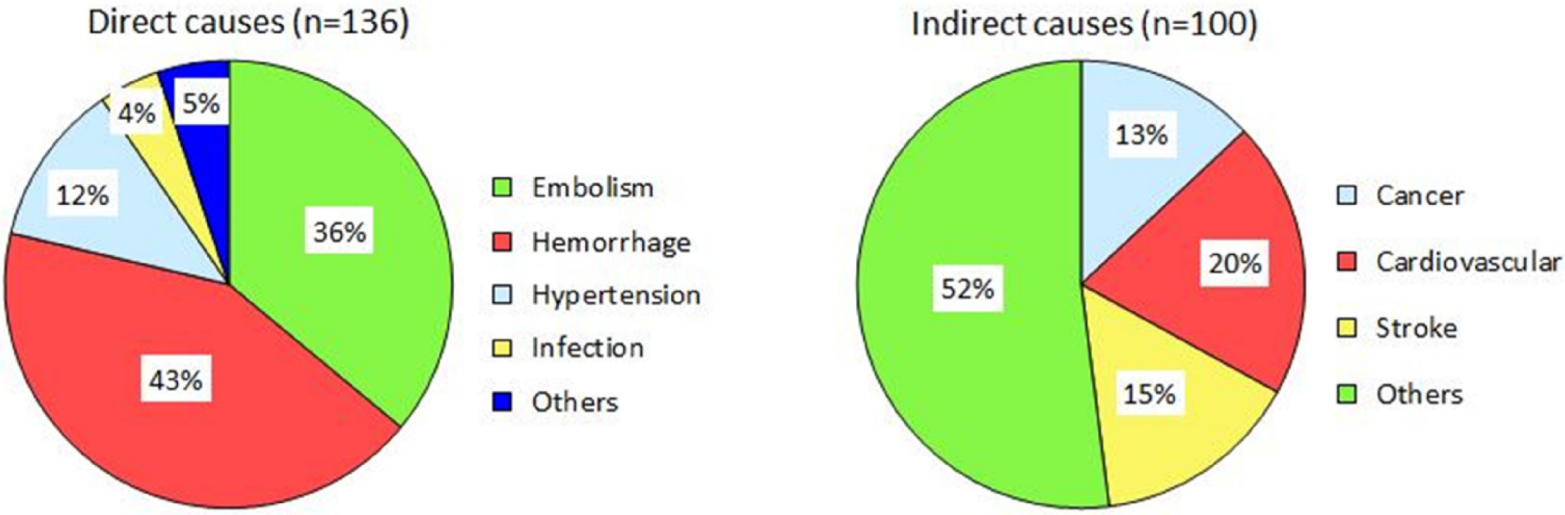
Causes of maternal death in Taiwan for 2004–2011.

Approximately two-fifths of maternal deaths were attributed to direct causes. Amniotic fluid embolism and postpartum hemorrhage were the two leading direct causes of maternal death. Cardiovascular diseases and stroke were the two main indirect causes of maternal death (Fig 4). The numbers of maternal deaths according to cause, maternal age, and year are listed in Table 3. No significant changes in distribution of causes of maternal deaths by maternal age between 2004–2007 and 2008–2011. Women aged 15–29 years had relatively lower proportion in dying from direct causes (amniotic fluid embolism and obstetric hemorrhage) compared with their counterpart older women.

**Table 3:** Causes of maternal death according to age and year for 2004–2011 in Taiwan.

	2004–07		2008–11		2004–11	
	No.	%	No.	%	No.	%
**15–29 years old**						
**Embolism**	7	15.6	4	20.0	11	16.9
**Hemorrhage**	8	17.8	3	15.0	11	16.9
**Others**	30	66.7	13	65.0	43	66.2
**Total**	45	100.0	20	100.0	65	100.0
**30–34 years old**						
**Embolism**	10	20.4	10	25.6	20	22.7
**Hemorrhage**	17	34.7	9	23.1	26	29.5
**Others**	22	44.9	20	51.3	42	47.7
**Total**	49	100.0	39	100.0	88	100.0
**35–49 years old**						
**Embolism**	8	18.2	10	25.6	18	21.7
**Hemorrhage**	13	29.5	8	20.5	21	25.3
**Others**	23	52.3	21	53.8	44	53.0
**Total**	44	100.0	39	100.0	83	100.0
**15–49 years old**						
**Embolism**	25	18.1	24	24.5	49	20.8
**Hemorrhage**	38	27.5	20	20.4	58	24.6
**Others**	75	54.3	54	55.1	129	54.7
**Total**	138	100.0	98	100.0	236	100.0

## Discussion

In this nationwide population-based data linkage study, we observed that approximately two-thirds of identified maternal deaths were not recorded in the original officially published mortality data. The MMR according to linked data declined between 2004 and 2011 for all groups of reproductive-age women. However, an increase in the MMR and number of deaths caused by amniotic fluid embolism and indirect causes were observed for women aged 15–29 years from 2008–2009 to 2010–2011. This change might be due to a lower number of events.

The underreporting rate estimated in this study was 57%, which is similar to that estimated in previous studies [3–15]; however, the rate was 1.5 times higher than that estimated 20 years ago in Taiwan (i.e., 37%) [23]. One possible reason for the discrepancy between the two estimated rates could be that different evaluation methods were used. In Koa et al, public health nurses retrospectively inquired families of deceased patients to determine whether the deaths were related to pregnancy, and, on the basis of the collected relevant information, the causes of maternal death were revised [23]. In this study, we linked four administrative data sets to obtain a comprehensive estimation of the MMR.

Another reason for the discrepancy between the two estimated rates was that many women with fatal pregnancy-related complications were treated in intensive care units for a certain period before death. The physicians of the intensive care units who issued the death certificates might have unintentionally omitted mentioning pregnancy on the death certificate. The third reason for the discrepancy was that physicians in the 2000s in Taiwan were more prone to intentionally forgot reporting pregnancy status on the death certificates than their counterparts in the 1980s because the levels of litigation regarding maternal death were higher in the 2000s. Karimian-Teherani et al suggested that countries with lower levels of litigation and higher rates of autopsy for forensic reasons were associated with a lower underreporting rate of maternal death because the doctors readily mentioned the pregnancy status of the deceased women [9].

To tackle the problem of underreporting of the MMR in the officially published mortality data, numerous countries have conducted routine surveillance linking different data sets to estimate the MMR accurately and to monitor the health status of reproductive-age women [3–15]. In the United States, questions about the temporal relationship of death with pregnancy were added in the 2003 Revision of the U.S. Standard Certificate of Death, which resulted in an increase in the reporting of pregnancy-related deaths [24]. Similar questions have been added to the 2014 version of the standard death certificate in Taiwan. We expect that the underreporting rate of the MMR according to the officially published mortality data will be reduced in the near future.

According to the estimation of the Global Burden of Disease Study, the MMRs of Taiwan were 17.1 in 2000, 13.5 in 2003, and 12.2 in 2011 [25,26]. The trend in these studies was similar to that in the current study; that is, 18.4 in 2004–2005, 17.3 in 2006–2007, 12.5 in 2008–2009, and 12.6 in 2010–2011. The annual percentage of change in the MMR in Taiwan was −4.7% between 1990 and 2003 and −5.3% between 2003 and 2013 [26]. The estimated annual percentage of change in the MMR in this study was −3.9% between 2004 and 2011. The degree of MMR reduction was higher in Taiwan than in Japan (–2.9%; the MMR was 8.2 in 2003 and 6.1 in 2013) and South Korea (–2.6%; the MMR was 15.4 in 2003 and 12.0 in 2013) [26].

Regarding the causes of maternal death, amniotic fluid embolism was the leading cause of maternal death followed by postpartum hemorrhage, according to the findings of the current study. The leading cause of maternal death was embolism in France [3], Canada [5], New York City, the United States [8], and the United Kingdom [13], whereas it was hypertension-related disorder in the Netherlands [4], North Carolina, the United States [7], and Jamaica [15], and postpartum hemorrhage in Austria [9] and Jordan [14]. An increase in the number of maternal deaths caused by amniotic fluid embolism and indirect causes from 2008–2009 to 2010–2011 was observed among women aged 15–29 years. Kassebaum et al reported that the increase in other direct, indirect, and late causes of maternal death was consistent, with a global epidemiological transition [26]. In Taiwan, 7% of live births were babies born to immigrant mothers (mainly from Southeast Asia and China); however, the babies born to married immigrant mothers in Taiwan had favorable neonatal outcomes compared with those born to permanent resident Taiwanese mothers [27].

This study had several limitations. First, despite having an ID in each data set, some maternal deaths could not be identified because of errors in transcription, a common problem encountered in using administrative data sets. Second, many women with severe obstetric complications were treated in intensive care units and died more than 42 days after delivery (late pregnancy death according to the ICD-10 codes); hence, these cases were not included in this study. Third, we could not further analyze the causes of maternal death by using the ICD codes because the results based on few cases (less than three cases) cannot be publicly revealed.

## Conclusions

Through the linkage of four nationwide administrative data sets, this study indicated that officially published mortality data had 57% fewer maternal deaths reported than the linked data. We suggest that routine surveillance of maternal mortality by using sophisticated methods is necessary to monitor the health status of reproductive-age women. Furthermore, a comprehensive review of maternal deaths is necessary to explore the possible preventive factors for designing relevant prevention programs [28,29].
